# Electromagnetic stimulation increases mitochondrial function in osteogenic cells and promotes bone fracture repair

**DOI:** 10.1038/s41598-021-98625-1

**Published:** 2021-09-27

**Authors:** Alex M. Hollenberg, Aric Huber, Charles O. Smith, Roman A. Eliseev

**Affiliations:** 1 Center for Musculoskeletal Research, University of Rochester School of Medicine & Dentistry, Rochester, NY USA; 2 University of Rochester Medical Center, 601 Elmwood Ave, Rm 1-8541, Rochester, NY 14642 USA

**Keywords:** Cell biology, Stem cells

## Abstract

Bone fracture is a growing public health burden and there is a clinical need for non-invasive therapies to aid in the fracture healing process. Previous studies have demonstrated the utility of electromagnetic (EM) fields in promoting bone repair; however, its underlying mechanism of action is unclear. Interestingly, there is a growing body of literature describing positive effects of an EM field on mitochondria. In our own work, we have previously demonstrated that differentiation of osteoprogenitors into osteoblasts involves activation of mitochondrial oxidative phosphorylation (OxPhos). Therefore, it was reasonable to propose that EM field therapy exerts bone anabolic effects via stimulation of mitochondrial OxPhos. In this study, we show that application of a low intensity constant EM field source on osteogenic cells in vitro resulted in increased mitochondrial membrane potential and respiratory complex I activity and induced osteogenic differentiation. In the presence of mitochondrial inhibitor antimycin A, the osteoinductive effect was reversed, confirming that this effect was mediated via increased OxPhos activity. Using a mouse tibial bone fracture model in vivo, we show that application of a low intensity constant EM field source enhanced fracture repair via improved biomechanical properties and increased callus bone mineralization. Overall, this study provides supporting evidence that EM field therapy promotes bone fracture repair through mitochondrial OxPhos activation.

## Introduction

Bone fracture is a growing public health burden, with the incidence expected to rise as the world population ages^1,2^. In the United States, bone fracture is the most common musculoskeletal condition requiring hospitalization among Medicare patients (≥ 65 years of age) as a consequence of increased fall risk, osteoporosis, and bone fragility^3^. While most fractures heal without complications, approximately 5–10% result in delayed healing or nonunion^4,5^. The etiology of nonunion is not fully understood, but important systemic risk factors include smoking, diabetes, and cachexia^6^. Local factors, including inadequate fixation and poor vascularity, are also recognized as contributors to delayed fracture repair. Nonunions are currently managed definitively with surgery, however there remains a clinical need for non-invasive therapies to aid in the fracture healing process.

One therapeutic approach to improve bone healing is the use of electromagnetic (EM) field stimulation. First published in 1974, Basset et al. suggested that EM fields may promote bone formation and nonunion repair^7^. Many subsequent studies have confirmed the osteogenic potential of pulsed EM fields (PEMFs), which is currently an approved therapy for nonunion fractures, congenital pseudoarthrosis, osteoporosis, and failed spinal fusions^8–11^. Several studies have established that PEMF therapy in vitro promotes bone formation by increasing osteoblast proliferation and expression of osteoblast marker genes (e.g., *RUNX2/CBFA1, ALP*)^12–14^, while also suppressing bone resorption activity by osteoclasts^15^. In addition, PEMF therapy has been shown to stimulate differentiation of human bone marrow-derived stromal cells (BMSCs) into osteoblasts, thereby enhancing mineralization^16^. Although several cellular mechanisms by which PEMF regulates these osteogenic effects have been proposed^17–23^, our current understanding remains limited.

Interestingly, there is a growing body of literature describing positive effects of an EM field on mitochondria^24–27^. In our own work, we have previously shown that differentiation of osteoprogenitors into osteoblasts is an energy dependent process that requires activation of mitochondrial oxidative phosphorylation (OxPhos)^28,29^. We have also helped to establish that in the setting of mitochondrial dysfunction, osteoblast/osteocyte function and bone maintenance is impaired^30^. Furthermore, we have reported that improving mitochondrial function via inhibition of the mitochondrial permeability transition pore can improve bone fracture repair^31^. During the fracture healing process, an important early step is the recruitment, proliferation, and differentiation of osteoprogenitors at the fracture site^5,32,33^. As such, it is conceivable that EM field therapy promotes osteogenic differentiation and bone anabolism by optimizing mitochondrial function in osteoprogenitors and osteoblasts during fracture healing.

In this study, we tested the hypothesis that EM field stimulation can promote mitochondrial function in osteogenic cells, and thereby promote osteogenesis and improve fracture healing in a mouse tibial bone fracture model.

## Results

### EM field stimulation induces mitochondrial OxPhos in osteogenic cells in vitro

**Figure 1:**
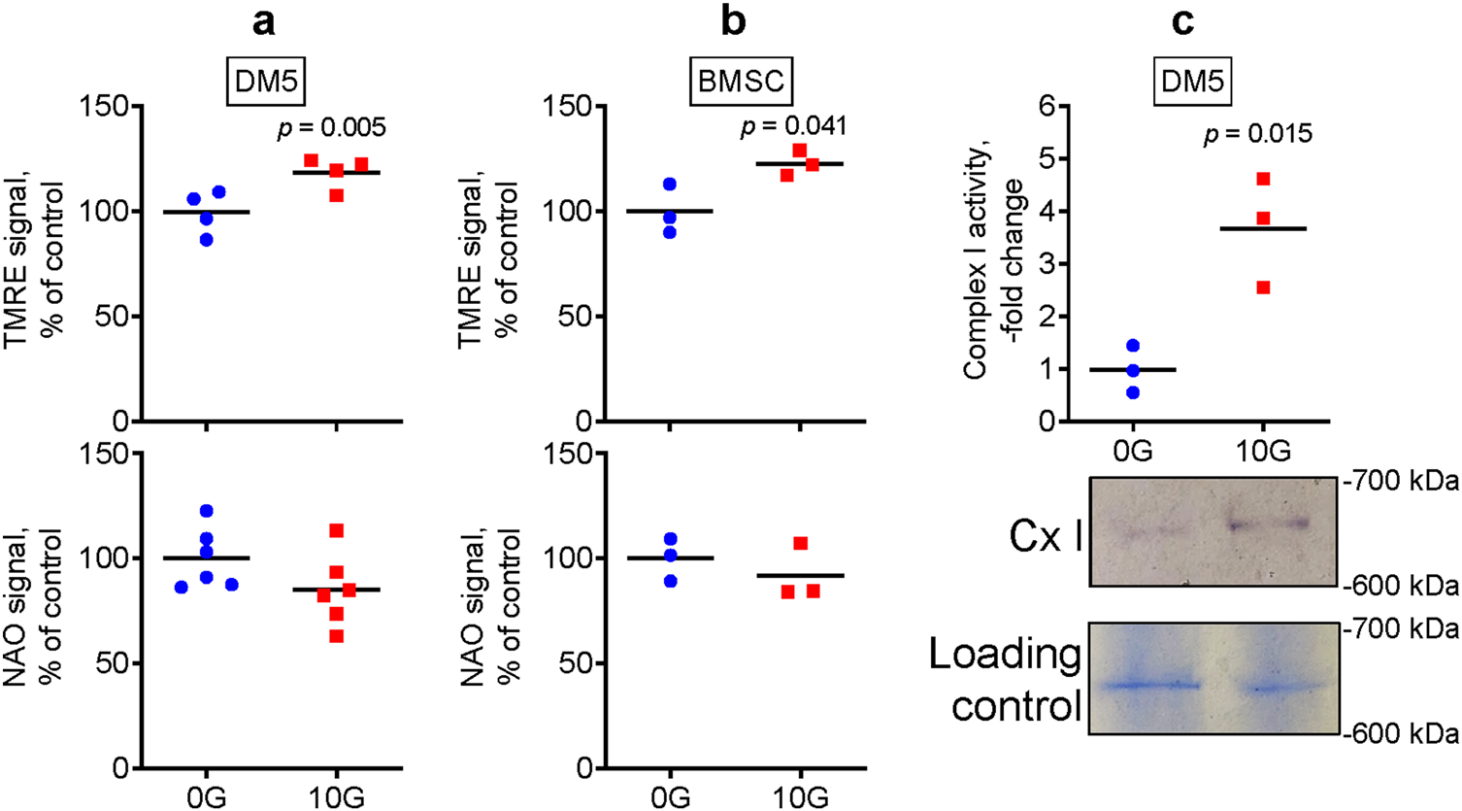
Electromagnetic field stimulation induces mitochondrial OxPhos in osteogenic cells in vitro. Osteogenic DM5 cells (**a**) or human BMSCs (**b**) were exposed to either an electromagnetic field at 10 Gauss (G) or left untreated (0G control). On day 4, cells were lifted and stained with either mitochondrial membrane potential probe tetramethyl rhodamine ester (TMRE) or mitochondrial mass probe nonyl acridine orange (NAO), as described in detail in the Methods. Fluorescence was assayed with flow cytometry; (**c**) DM5 cell lysates were run on a non-denaturing clear-native gel. Gels were exposed to enzymatic colorimetric mitochondrial respiratory complex I (Cx I) assay. Cx I signal was measured and normalized to loading controls stained with Coomassie Blue. A representative gel image is shown below the graph. Uncropped images are shown in Supplementary Fig. S1a. Plots show actual data points and calculated means. *P* value was determined via the *t*-test.

Fracture healing is a multi-stage process that involves a variety of cells, including immune cells, endothelial cells, and osteoprogenitors^4^. At the fracture site, osteoprogenitors differentiate into bone forming osteoblasts^5^. We and others have previously reported that BMSCs upregulate mitochondrial OxPhos during osteoblast differentiation, and this upregulation promotes bone forming function^28^. Therefore, we first tested whether EM field stimulation can induce mitochondrial activity in osteogenic DM5 cells and BMSCs. Our method of choice to analyze mitochondrial activity was a mitochondrial membrane potential measurement using tetramethyl rhodamine ester (TMRE) staining, because it can be applied during the exposure and then immediately assayed using flow cytometry. Our second method was a mitochondrial respiratory complexes I–IV enzymatic activity assay. We preferred these methods over other available methods, such as the Seahorse technology-based oxygen consumption assay, because these methods do not require prolonged monitoring of cells post-EM field exposure. During such a prolonged monitoring in a Seahorse machine, the EM effect may weaken or disappear. Using TMRE fluorescent staining, we found that exposing osteogenic cells to an EM field at 10 Gauss (G) for 4 days caused a significant increase in mitochondrial membrane potential as compared to cells without EM field stimulation (0G control, Fig. 1a,b). Mitochondrial mass was also measured simultaneously using nonyl acridine orange (NAO) staining, which showed no significant difference in NAO signal between the exposed and nonexposed control cells (Fig. 1a,b, bottom graphs). We also performed an EM dose response experiment using 2.5G, 5G, 10G, 15G, and 20G, and found that 10G was sufficient to induce the observed effects, whereas 15G and 20G did not significantly change it (not shown). In addition, exposed DM5 cells displayed significantly upregulated mitochondrial respiratory complex I enzymatic activity as compared to the nonexposed control cells (Fig. 1c, Suppl Fig. S1a). Complex II, III, and IV activities remained unchanged following EM field exposure (Suppl Fig. S1b). We also measured ATP content after exposure to EM field stimulation, which showed no significant changes (DM5: 107 ± 11% of control, *p* = 0.127 via *t*-test with n = 3; BMSC: 112 ± 9% of control, *p* = 0.086 via *t*-test with n = 3). Lack of changes in ATP content can likely be explained by the fact that while higher mitochondrial OxPhos activity leads to higher ATP production, increased cellular activity leads to increased ATP consumption. Altogether, these results support the ability of EM field stimulation to upregulate mitochondrial function and OxPhos activity in osteogenic cells in vitro.

### EM field stimulation promotes bone-forming activity of osteogenic cells in vitro

**Figure 2:**
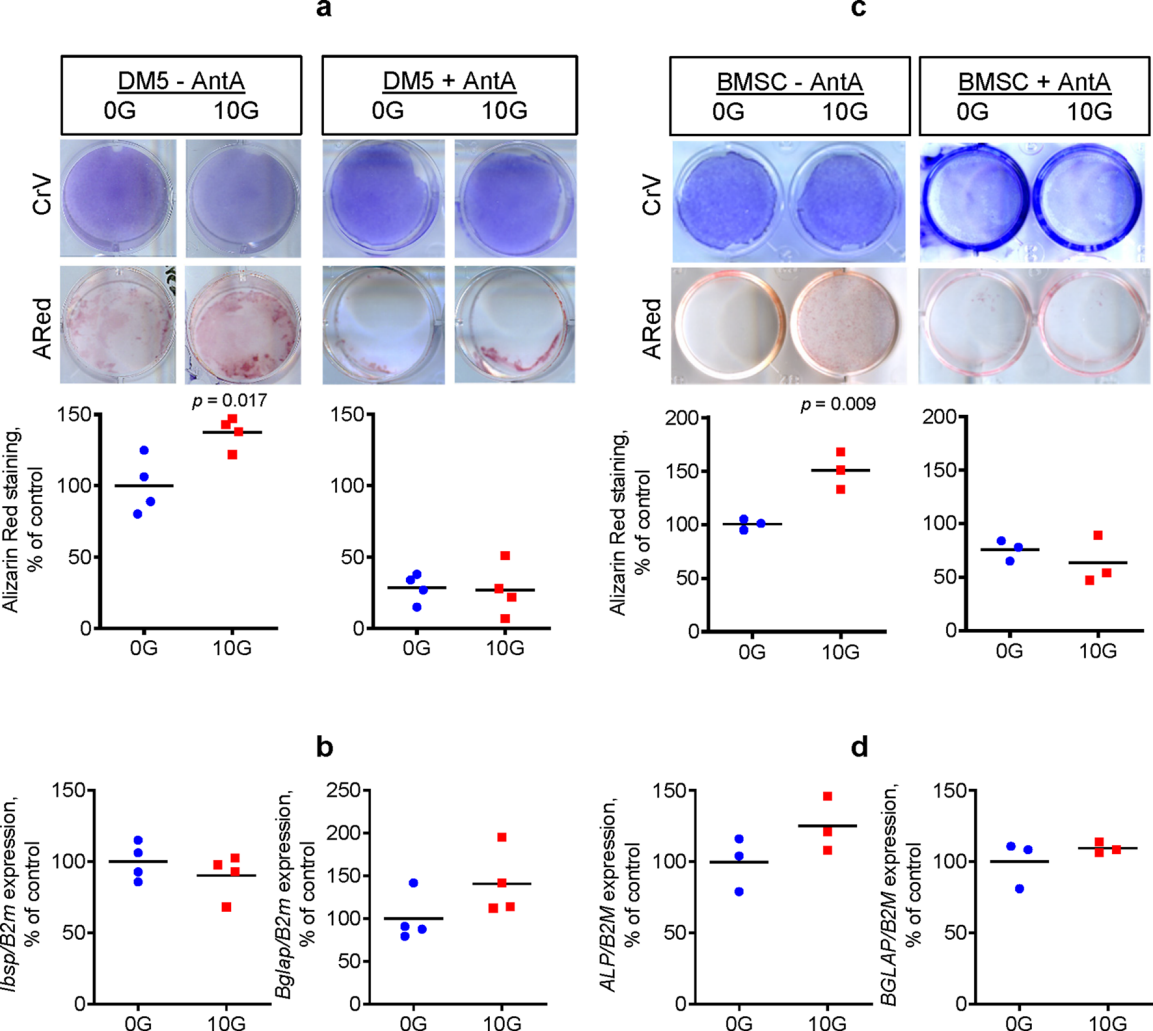
Electromagnetic field stimulation promotes bone-forming activity of osteogenic cells in vitro. Mouse osteogenic DM5 cells (**a**, **b**) or human BMSCs (**c**, **d**) were exposed to an electromagnetic field at 10 Gauss (G) or left untreated (0G control) in the absence or presence of antimycin A (AntA) at 0.1 mM. On day 4, cells were stained with Alizarin Red (ARed) to assess calcium deposition or with Crystal Violet (CrV) to assess proliferation (**a**, **c**). Plates were scanned and ARed and CrV staining intensities were measured with ImageJ software. ARed signal was normalized to CrV signal. Cells were also collected for RNA extraction and real-time RT-PCR analysis was performed for the indicated genes of interest normalized to *B2m* (**b**, **d**). Images are representatives of 4 (DM5) or 3 (human BMSC). Plots show actual data points and calculated means. *P* value was determined via the *t*-test.

Previous studies have established that upregulated mitochondrial function increases osteogenic potential in osteoprogenitors^28,29,34,35^. After determining in the previous experiment (Fig. 1) that EM field stimulation can upregulate mitochondrial OxPhos function, we next investigated whether EM field exposure enhances osteogenesis and osteoblast function. Using Alizarin Red (ARed) staining that detects calcium deposition due to osteoblast activity, we found that both DM5 cells and human BMSCs exposed to an EM field at 10G had significantly increased in vitro calcium deposition as compared to the nonexposed cells (Fig. 2a,c). Cellular proliferation for both cell types were not affected by EM field exposure, measured by crystal violet (CrV) staining. To confirm that the effect of EM field stimulation on osteogenic function was mediated via mitochondrial OxPhos and complex I activity induction, we first exposed cells in the presence of mitochondrial respiratory complex I inhibitor rotenone. However, rotenone, even at low concentrations, was toxic to both cell types (not shown). Therefore, we modified our approach and used a compound that affects the mitochondrial respiratory chain downstream from complex I, in particular, complex III inhibitor antimycin A (AntA). We observed that AntA reversed the osteoinductive effect of EM field exposure (Fig. 2a,c). In DM5 cells, mRNA expression of early osteoblast marker *Ibsp* was not significantly changed after 4 days of EM field treatment, while mRNA expression of late osteoblast marker *Bglap* was increased, but not significantly (Fig. 2b). In human BMSCs, both osteoblast markers *ALP* and *BGLAP* were induced by EM field exposure, but not significantly (Fig. 2d). Thus, the osteogenic effect was likely not associated with changes in gene expression, but rather associated with changes in cellular activity due to increased bioenergetics. It is worth mentioning that while other studies have reported significant increases in osteoblast gene markers following EM field stimulation^12–14^, this difference may be related to the intensity of the EM field. In our study, we used a very low intensity constant EM field source, which is in contrast to the much higher intensities generated from PEMF therapy. Also, it should be noted that when compared to primary BMSCs, DM5 cells are more primed towards an osteogenic lineage as indicated by the presence of ARed staining at baseline (compare panels a and c in Fig. 2) and higher alkaline phosphatase staining (not shown). Treatment with AntA resulted in decreased ARed staining in the control DM5 cells, which is expected because according to our previous reports^28,36^, OxPhos activity is required for osteoblast maturation. In human BMSCs, which are less differentiated than DM5 cells and show lower basal ARed staining, AntA prevented the EM-induced increase in staining. Altogether, these results provide evidence that EM field stimulation promotes bone forming activity in osteogenic cells in vitro through enhanced mitochondrial function.

### EM field stimulation promotes bone formation during mouse tibial fracture repair

**Figure 3:**
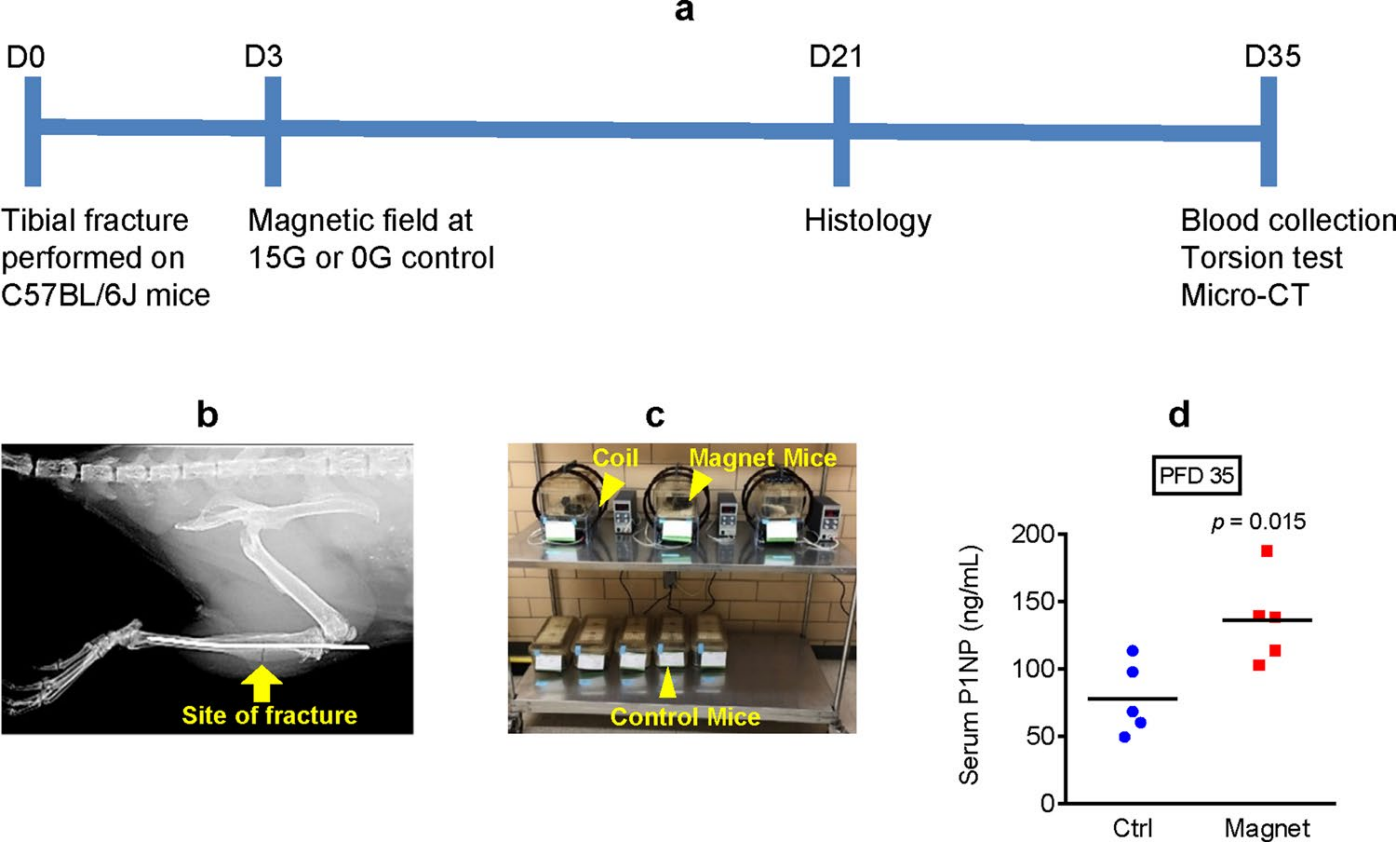
Electromagnetic field stimulation promotes bone formation during mouse tibial fracture repair. (**a**) Diagram outlining the experimental setup; (**b**) X-Ray image of tibia that underwent fracture with intramedullary pin inserted; (**c**) Picture of mice exposed and not exposed to the electromagnetic field; (**d**) Serum bone formation marker propeptide of type I procollagen (P1NP) was measured via ELISA on post-fracture day (PFD) 35. Plot shows actual data points and calculated means. *P* value was determined via the *t*-test.

After determining that EM field exposure has enhancing mitochondrial and osteogenic effects in mouse DM5 cells and human primary BMSCs in vitro, our next step was to determine whether these effects can be translated in vivo to promote bone fracture healing. To test this, unilateral mouse tibial fractures were performed on skeletally mature 3-month-old C57BL/6J male mice and then subjected to either EM field exposure at 15G beginning on post-fracture day (PFD) 3 or no exposure (0G control). We used a slightly higher EM field strength as compared to our in vitro experiments to account for possible loss of signal in animal tissue. We also used male mice only to avoid potential confounding effects of estrogen in female mice, as estrogen is known to stimulate mitochondrial function^37,38^. On PFD 21 and 35, experimental and control mice were sacrificed and their fractured tibiae were isolated and analyzed, as illustrated in Fig. 3a. Figure 3b is a representative X-Ray image of a tibial fracture with an intramedullary pin inserted on PFD 0. Figure 3c illustrates the experimental setup, with mice exposed to an EM field inside the induction coils and nonexposed control mice outside the coils. To determine whether EM field exposure promotes bone formation during fracture repair, serum was collected on PFD 35 and propeptide of type I procollagen (P1NP) levels were measured. P1NP is produced by amino-terminal and carboxy-terminal splicing of type 1 procollagen in osteoblasts, and therefore is a specific marker of bone formation^39^. In comparison to the nonexposed control mice, the exposed mice had significantly higher serum P1NP levels (Fig. 3d). This finding supports the hypothesis that EM field therapy can promote bone anabolism during fracture healing.

### EM field stimulation improves biomechanical properties after tibial fracture repair in mice

**Figure 4:**
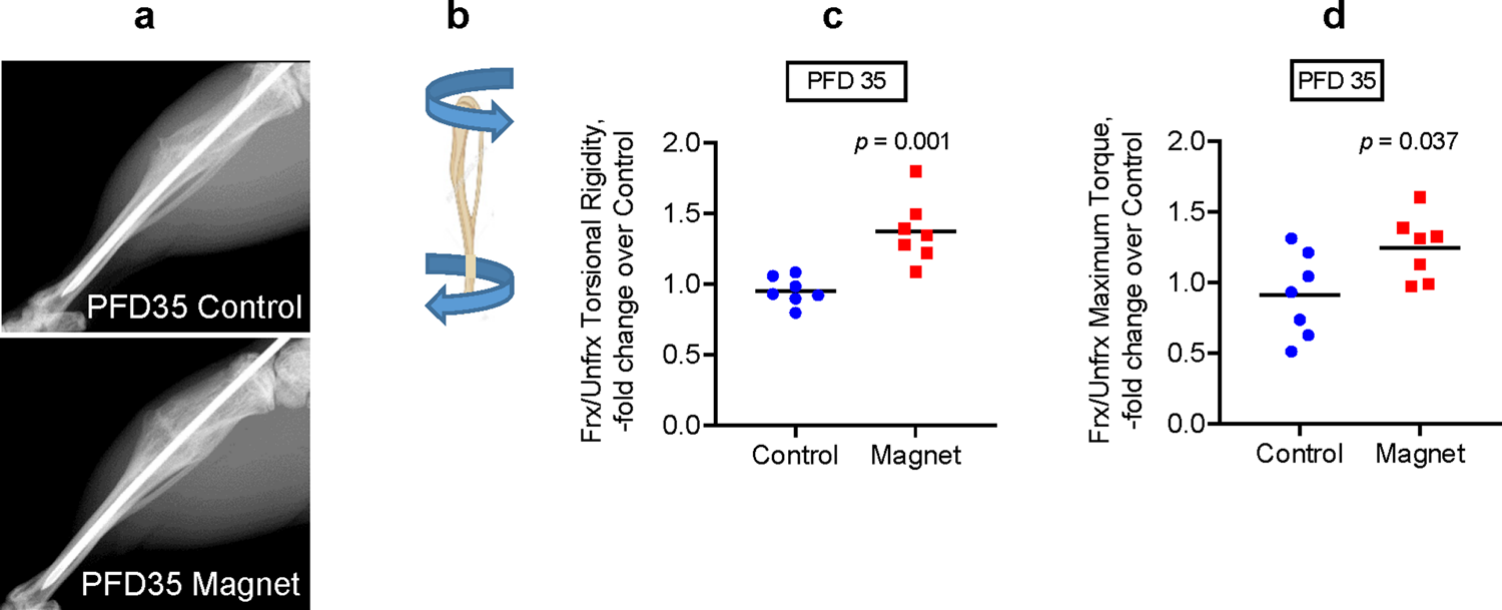
Electromagnetic field stimulation improves biomechanical properties after tibial fracture repair in mice. (**a**) X-Ray images of repaired tibiae on post-fracture day (PFD) 35; (**b**) Diagram illustrating the torsion test performed on tibia; (**c**) Torsional Rigidity values derived from the torsion test; (**d**) Maximum Torque values derived from the torsion test. Plots show actual data points and calculated means. Both fractured (Frx) and unfractured (Unfrx) tibiae were tested. Values in Frx bone are normalized to values in Unfrx bone to account for systemic changes after exposure to the electromagnetic field. Plots show actual data points and calculated means. *P* value was determined via the *t*-test.

Next, we performed biomechanical testing to compare the quality of repaired bone between the exposed and nonexposed control mice. Figure 4a is a representative X-Ray image on PFD 35 showing that the callus size and bony bridge formation were similar between the control and experimental mice. Mice were sacrificed and the fractured and contralateral unfractured tibiae on PFD 35 were isolated and subjected to torsion testing, as illustrated in Fig. 4b. Mice that were exposed to the EM field had tibiae that demonstrated significantly enhanced biomechanical properties (torsional rigidity and maximum torque) as compared to the nonexposed control mice (Fig. 4c,d). Of note, we normalized data from fractured tibiae to data from contralateral unfractured tibiae to account for possible systemic effects on bone since the whole mouse was exposed to an EM field and not just the fractured leg. This data provides evidence that EM field therapy can improve the biomechanical strength of bone after fracture repair.

### EM field stimulation increases callus mineralization during tibial fracture repair in mice

**Figure 5:**
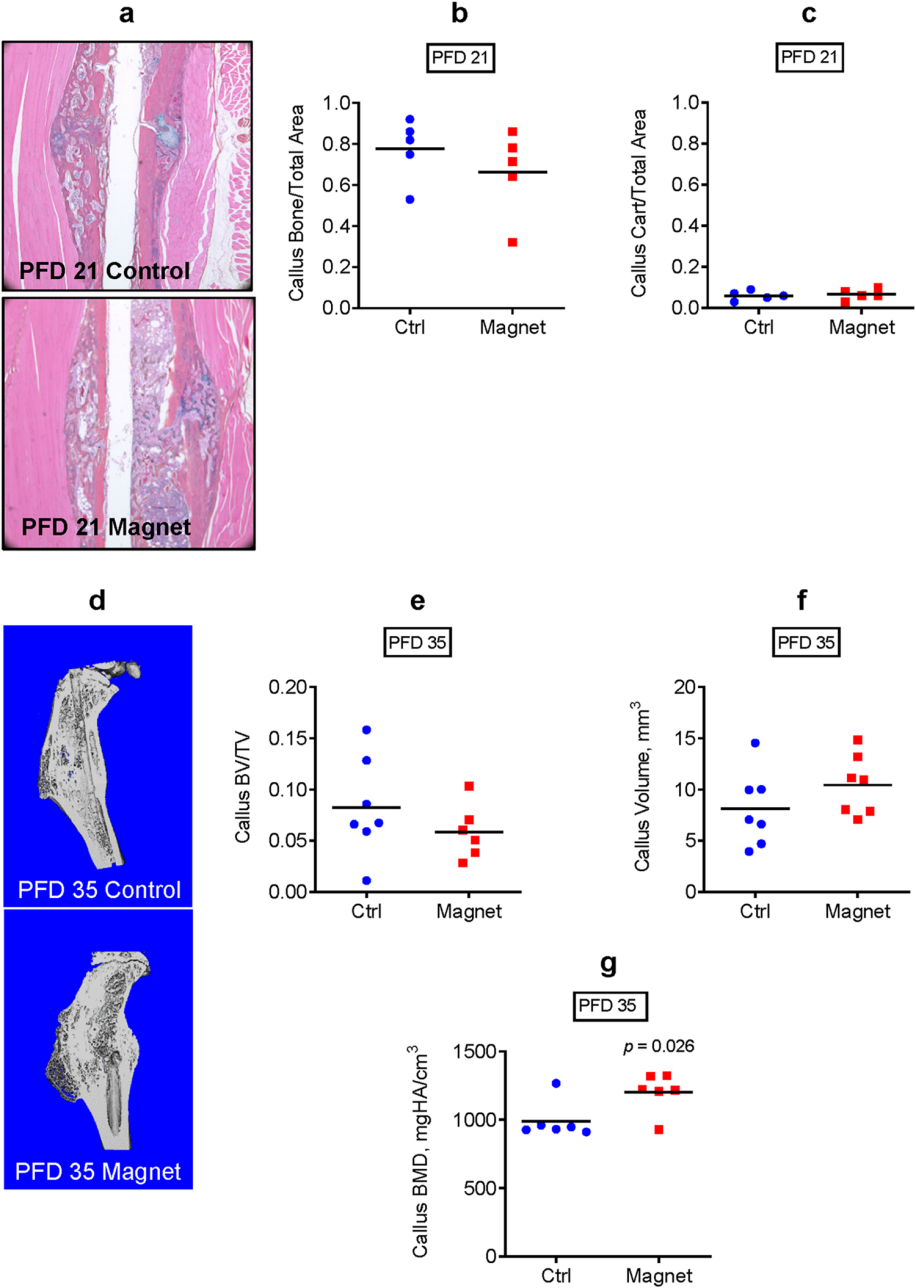
Electromagnetic field stimulation increases callus mineralization during tibial fracture repair in mice. (**a**) Histology of tibial fracture callus on post-fracture day (PFD) 21 and histomorphometric analyses of (**b**) callus bone vs total area and (**c**) callus cartilage vs total area; (**d**) Sagittal sections of 3D-reconstructed images from micro-CT analysis on PFD 35; (**e**) Total callus volume measured with micro-CT; (**f**) Bony callus fraction (BV/TV) measured with micro-CT; (**g**) Callus bone mineral density (BMD) measured with micro-CT. Plots show actual data points and calculated means. *P* value was determined via the *t*-test.

To investigate whether the enhanced biomechanical strength in the EM field-exposed repaired tibiae was associated with callus structural changes, we first performed histomorphometric analysis of the fractured callus on PFD 21, an intermediate time-point in the tibial fracture repair process in C57BL/6 J mice (Fig. 5a). As shown in Fig. 5b,c, this assay did not detect any significant changes in either callus bone or callus cartilage content. We then performed whole callus micro-CT on PFD 35 to assess bony callus formation. Figure 5d is a representative sagittal section of a 3D-reconstructed image from micro-CT. Our results indicate that there were no significant differences in total callus volume (Fig. 5e) or bony callus fraction (BV/TV) (Fig. 5f) between the exposed and nonexposed mice, which is consistent with the results from the histomorphometric analysis. However, the exposed mice did have significantly elevated callus bone mineral density (BMD, Fig. 5g), likely contributing to the enhanced biomechanical properties observed in Fig. 4.

## Discussion

Mitochondrial bioenergetics is gaining important recognition in the bone field. Our group and others have previously demonstrated the role of mitochondrial OxPhos during osteoblast differentiation^28,29,35,40,41^. We have also shown that protecting mitochondrial function by inhibiting permeability transition pore activity decreases age-related bone loss^30^. Most recently, using a proof-of-concept strategy to upregulate mitochondrial OxPhos activity through systemic administration of a glycolytic inhibitor, we were able to improve BMD, cortical bone architecture, and bone biomechanical properties in both young and old mice^42^.

In light of these previous findings, we sought to explore other therapeutic avenues to upregulate mitochondrial OxPhos activity, and thereby improve bone fracture repair. In this study, we demonstrate that applying a constant EM field source at 10G on osteogenic DM5 cells and human BMSCs generated increased mitochondrial membrane potential, and this increase was likely related to stimulation of mitochondrial respiratory complex I activity. Next, we show that EM field stimulation at 10G induced osteogenic activity in DM5 cells and human BMSCs. In the presence of mitochondrial respiratory complex III inhibitor AntA, this osteoinductive effect was reversed, confirming that the effect of EM field stimulation on osteogenic function was mediated via increased mitochondrial OxPhos activity. Lastly, we show that applying a constant EM field source at 15G was sufficient to enhance tibial fracture repair in mice via improved biomechanical properties and increased callus bone mineralization. Overall, these results provide support for the hypothesis that EM field therapy promotes bone fracture repair through mitochondrial OxPhos activation.

EM field therapy has been shown for many years to be clinically beneficial in the treatment of impaired fracture healing^11,43,44^, however its application has remained limited likely due to an uncertain underlying mechanism of action. With regards to possible mechanisms of action, it was assumed that the mitochondria might represent a uniquely sensitive organelle to EM field stimulation given the high protein content on its inner membrane surface^45^. Many of these proteins are iron/sulfur-containing, and shuttle electrons across the membrane. Indeed, the application of EM fields has been demonstrated to directly impact respiration rates of liver mitochondria^24^, ATP linked respiration and proton leak in lymphocytes^25^, and mitochondrial membrane potential of numerous cancer cell lines^26^. Additionally, EM field therapy has been shown to modulate cancer cell metabolism by causing significant changes in intermediates of the glycolysis and tricarboxylic acid cycle pathways^46^.

Recently, it was also proposed that EM fields may activate transmembrane receptors, initiating downstream signal cascades involved in osteoblast proliferation, differentiation, and mineralization^47–49^. Yumoto et al. found that EM wave irradiation of osteoblastic MC3T3-E1 cells significantly enhanced osteoblast proliferation, as well as upregulated various growth factors, including vascular endothelial growth factor and platelet-derived growth factor, via induction of ERK1/2 and p38 MAPK pathways^21^. Ehnert et al. supported these findings by reporting a strong activation of the ERK1/2 signaling pathway in primary human osteoblasts after extremely low frequency PEMF (ELF-PEMF) treatment, resulting in significantly increased total protein content, alkaline phosphatase activity, and matrix mineralization^22^. Interestingly, these effects were also accompanied by significantly upregulated mitochondrial activity. Upon further investigation, Ehnert et al. proposed that EM fields induce mitochondrial production of nontoxic amounts of reactive oxygen species (ROS), which then initiate an antioxidant stress response essential for osteoblast differentiation^23^. Supporting these findings, Chen et al. demonstrated a coordinated regulation of mitochondrial biogenesis and antioxidant enzymes that occur synergistically during osteogenic differentiation of human BMSCs^35^. In the setting of mitochondrial dysfunction, it has been established that pathological levels of intracellular ROS accumulate and subsequently result in opening of the mitochondrial permeability transition pore, leading to mitochondrial swelling, decreased ATP production, and ultimately osteoblast dysfunction^50,51^. We have previously shown that protecting mitochondria from excessive permeability transition pore opening via inhibition of cyclophilin D improves bone quality and fracture repair^30,31^. Altogether, these studies in conjunction with our results highlight the mitochondria as not only playing a central role in bone homeostasis, but also as a potential downstream target of EM field therapy.

A noteworthy aspect of our study was our use of a low intensity constant EM field source, which was applied for 24 h per day for the duration of the experiment. This is in contrast to the frequently employed PEMF therapy, which generates a group of stationary high intensity pulses for intermittent short periods of time. Whereas PEMF therapy is applied several times per day, our low intensity constant EM field source is appropriate for a long-term use device. Future work is needed to determine how differences in frequencies and intensities of EM fields alter cellular metabolism and bone function. In addition, while current evidence suggests that EM field stimulation may offer clinical benefit in the treatment of impaired fracture healing, future well conducted and adequately powered randomized controlled clinical trials are needed to better inform clinical practice^52^. Overall, we demonstrate in this study that EM field therapy in vitro stimulates mitochondrial OxPhos activity via induction of mitochondrial respiratory complex I activity, and this promotes bone forming function of osteogenic cells. These results were translated in vivo to promote bone fracture repair.

## Materials and methods

### Materials

Chemicals were obtained from Sigma unless otherwise stated. Cell culture media and components were obtained from Gibco. The low intensity constant EM field source was generated using a Pair of Helmholtz Coils No 1000906 (3B Scientific) attached to the TekPower TP3005P power supply.

### Cell culture, EM field exposure, and staining

The osteogenic DM5 cell line derived from mouse bone marrow was a kind gift from Dr. Pamela Robey (National Institutes of Health). Human BMSCs were obtained from Lonza. Cells were cultured in DMEM media with 10% fetal bovine serum and 1% penicillin/streptomycin. To expose cells to an EM field at 10G, EM induction coils were placed inside the tissue culture incubator and cell culture plates were positioned inside the coils on custom made stands. EM field strength at this position was verified using a gaussmeter. Control cells (0G) were placed outside of the coils. After 4 days of EM field exposure, cells were stained with either ARed to assay for calcium deposition or with CrV to assay for proliferation. Stained wells were scanned and staining intensity was measured using ImageJ software (version 1.8.0, http://imagej.nih.gov/ij, National Institutes of Health). Measured ARed signal was normalized to CrV signal. A separate set of cells were collected for RNA isolation and gene expression analysis using real-time RT-PCR.

### Real-time RT-PCR

Total RNA was isolated using the RNeasy kit (Qiagen) and reverse transcribed into cDNA using the qScript cDNA synthesis kit (Quanta). CDNA was subjected to real-time RT-PCR. The primer pairs used for genes of interest are as follows: *Ibsp* (5′-AAT GGA GAC GGC GAT AGT-3′ and 5′-GAG TGC CGC TAA CTC AAA-3′), *Bglap* (5′-GAC CTC ACA GAT GCC AAG-3′ and 5′-CAA GCC ATA CTG GTC TGA TAG-3′), *B2m* (5′-AAT GGG AAG CCG AAC ATA C-3′ and 5′-CCA TAC TGG CAT GCT TAA CT-3′), *ALP* (5′-TGC AGT ACG AGC TGA ACA GGA ACA-3′ and 5′-TCC ACC AAA TGT GAA GAC GTG GGA-3′), and *B2M* (5′-CAG CAA GGA GTC TTT CTA-3′ and 5′-ACA TGT CTC GAT CCC ACT TAA-3′). Real time RT-PCR was performed in the RotorGene system (Qiagen) using SYBR Green (Quanta). The expression of genes of interest was normalized to expression of *B2m* (β-2 microglobulin).

### Mitochondrial membrane potential and mass assays using flow cytometry

Cells were stained with mitochondrial membrane potential probe TMRE at 20 nM and mitochondrial mass probe NAO at 100 nM. Since TMRE is a dynamic probe that may leak out of the mitochondria due to probe redistribution during washing, TMRE was present in the wash and assay buffer. TMRE and NAO signals were detected using flow cytometry at the University of Rochester Core Facility using BD Biosciences LSRII flow cytometer. TMRE signal was normalized to NAO signal to account for possible differences in mitochondrial mass.

### Cellular ATP assay

ATP levels were measured in cell lysates using the CellTiter-Glo luminescence kit (Roche) according to manufacturer’s instructions. Luminescence was measured in BioTek plate reader.

### Mitochondrial respiratory complexes I–IV activity assay

For complex I assay, cells were lysed in a buffer consisting of lauryl maltoside (1%), NaCl (50 mM), imidazole (50 mM), aminocaproic acid (2 mM) and EDTA (1 mM), pH 7.0 at 4 °C, and subjected to 4–10% gradient clear native polyacrylamide gel electrophoresis (CN PAGE). Complex I activity was determined as described previously by incubating gel in a buffer containing Tris–HCl at 5 mM (pH 7.4), NADH at 0.1 mg/mL, and nitroblue tetrazolium at 2.5 mg/mL and rocking overnight at room temperature^53^. Complex I monomer bands at 669 kDa were measured with densitometry using ImageJ software. Gel was later stained with Coomassie Blue total protein stain as a loading control. Complex I signal was normalized to Coomassie Blue signal.

Complex II, III, and IV activities in cell extracts were assayed enzymatically as described in our previous report using BioTek plate reader^54^. Briefly, for complex II activity, 0.05 mM ubiquinone-2, 1 µM rotenone, and 20 mM succinate were added to the sample diluted in a buffer made of 50 mM KPi, 0.1 mM EDTA, 45 µM 2,6-dichlorophenolindophenol, 1 mM KCN, and 2.5 mg/ml BSA. Color change of 2,6-dichlorophenolindophenol upon addition of 1 mM thenoyltrifluoroacetone was monitored at 600 nm. For complex III assay, production of reduced cytochrome c was monitored at 550 nm. The sample was added to a buffer that contained 50 mM KPi, 1 mM EDTA, 5 mM MgCl_2_, 20 mM KCN, 1 µM rotenone, 15 µM cytochrome c, and 15 µM ubiquinol. For complex IV assay, oxidation of 50 µM reduced cytochrome c by the sample was monitored at 550 nm in 10 mM KPi. The slope of the absorbance curve was used to determine the rate constant for cytochrome oxidase activity.

### Animal study approval

The study was carried out in compliance with the ARRIVE guidelines. Animal husbandry and experiments were performed in accordance with the Division of Laboratory Animal Medicine, University of Rochester, state and federal law, and National Institutes of Health policy. The University of Rochester Institutional Animal Care and Use Committee (IACUC) specifically approved this study.

### Experimental groups of mice

The C57BL/6J mouse strain was obtained from the Jackson Laboratory (RRID: JAX:000664). These mice have been shown to repair fractures more efficiently when compared to other mouse strains (e.g., C3H, DBA/2)^55^. Mice were housed at 23 °C on a 12-h light/dark cycle with free access to water and PicoLab Rodent Diet 20 (LabDiet #5053). The treatment groups were: seven 3-month-old male mice with tibial fracture exposed to a 0G EM field (control) and seven 3-month-old male mice exposed to a 15G EM field. EM therapy treatment began on PFD 3 until PFD 35.

### Tibia fracture

Mice were subjected to unilateral tibial fractures under anesthesia with ketamine/xylazine mixture (100 mg kg^−1^/10 mg kg^−1^) intraperitoneal injection. The depth of anesthesia was confirmed using a toe pinch reflex test. The right tibia was exposed, and a cut was made through the mid-diaphyseal shaft with a scalpel to simulate fracture. A 27 Gauge stainless steel pin was inserted intramedullary for stabilization. Each fracture was verified with an X-ray post-fracture. The left tibia served as the unfractured control. Mice were collected on PFD 35 for analyses and biomechanical testing.

### Serum P1NP assay

Blood was harvested before sacrifice and serum was prepared after centrifuging at 10,000 rpm for 10 min at 4 °C in a table-top centrifuge. The bone formation marker P1NP was quantified using an enzyme immunoassay from Immunodiagnostic Systems.

### Histology and histomorphometry

A cohort of mice undergoing tibia fracture were sacrificed on PFD 21. Calluses were formalin-fixed, decalcified in EDTA, and paraffin-embedded as described previously^31^. Five µm sections were cut at 3 levels of each sample and then stained with Alcian Blue/Hematoxylin/Orange G (ABH/OG) trichrome. Histomorphometry was performed using OsteoMeasure software and bone, cartilage, and total callus areas were measured at all 3 levels and averaged.

### Micro-CT of fractured and unfractured bones

Tibiae were imaged using VivaCT 40 tomograph (Scanco Medical). Scanco analysis software was utilized for volume quantification. Trabecular bone vs total volume (BV/TV), BMD, and cortical thickness were determined for unfractured tibiae, while callus total volume, callus BV/TV, and callus BMD were determined for fractured tibiae. Fracture groups subjected to an EM field were compared to control groups.

### Biomechanical torsion test

Following euthanasia, the fractured and unfractured tibiae were isolated and cleaned of excess soft tissue. Tibiae were stored at − 80 °C; and the intramedullary pins were removed prior to biomechanical testing. Tibiae were subjected to torsional strain. The ends of the tibias were cemented (Bosworth Company) in aluminum tube holders and tested using an EnduraTec TestBench system (Bose Corporation). The tibiae were tested until failure at a rate of 1°/sec. The torque data were plotted against rotational deformation to determine maximum torque and torsional rigidity.

### Statistical analyses

Sample size for experiments was determined via power analysis using the following formula: $\mathrm{n}=\frac{2({{\mathrm{Z}}_{\mathrm{a}}+{\mathrm{ Z}}_{1-\upbeta })}^{2\upsigma 2}}{{\Delta }^{2}}$ (n = sample size, α = type I error, β = type II error, Δ = effect size, σ = standard deviation, and Z is a constant) based on previous data and pilot studies. Data were analyzed using Prism GraphPad software (version 9.0.1). Mean values were calculated and the statistical significance (*P* < 0.05) was established using Student’s t-test based on normal spread of our data.

## Supplementary Information

Supplementary Legends.

Supplementary Figure S1.

## Data Availability

Original data files are available upon request.
