# Supplementary material for: Electromagnetic stimulation increases mitochondrial function in osteogenic cells and promotes bone fracture repair
Source: Sci Rep. 2021 Sep 27;11:19114. doi: 10.1038/s41598-021-98625-1 (PMC8476611; doi:10.1038/s41598-021-98625-1)
Supplement: Supplementary file 1 — Supplementary Legends. [file 41598_2021_98625_MOESM1_ESM.docx]

**Electromagnetic Stimulation Increases Mitochondrial Function in Osteogenic Cells and Promotes Bone Fracture Repair**

Alex M. Hollenberg^1^, Aric Huber^1^, Charles O. Smith^1^, Roman A. Eliseev^1,*^

**SUPPLEMENTARY INFORMATION**

Supplementary Figure S1 (related to Figure 1). **Mitochondrial respiratory complexes activity assay.** (**a**) Cell lysates were run on a non-denaturing clear-native gel and subjected to enzymatic colorimetric assay, as described in Methods. Shown are a representative (n = 3) uncropped developed clear-native gel image and loading control stained with Coomassie Blue; (**b**) enzymatic activities of respiratory complexes (Cx) II-IV were measured, as described in Methods. The data are expressed as percentage of activity in cells exposed to 10G EM field compared with control cells. Plots show actual data points and calculated means. *P* value was determined for each complex independently vs control cells via the *t*-test.
