# Supplementary figures and images for: Electromagnetic stimulation increases mitochondrial function in osteogenic cells and promotes bone fracture repair
Source: Sci Rep. 2021 Sep 27;11:19114. doi: 10.1038/s41598-021-98625-1 (PMC8476611; doi:10.1038/s41598-021-98625-1)

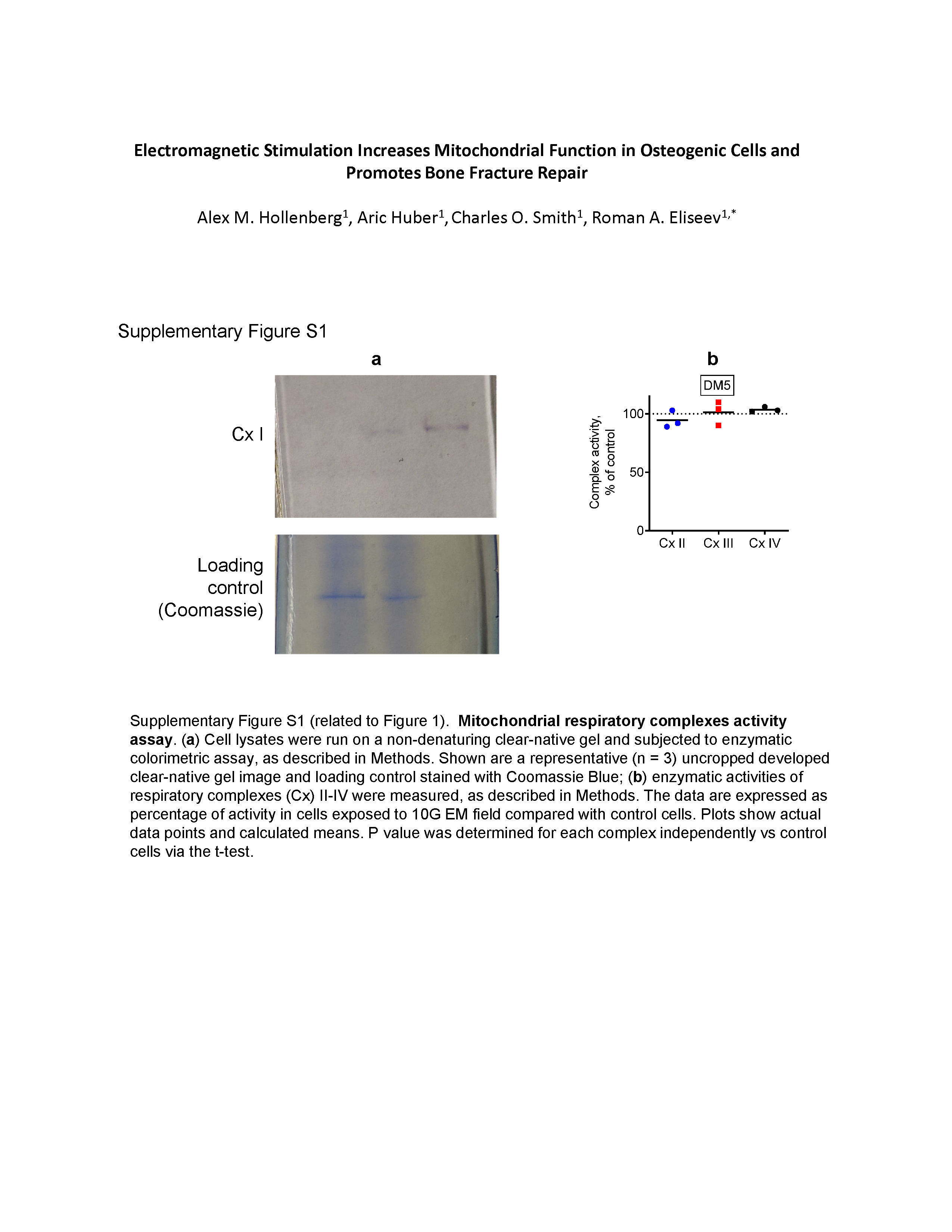

Supplement: Supplementary file 2 — Supplementary Figure S1. [file 41598_2021_98625_MOESM2_ESM.tif]
